# Supplementary material for: Ischemic stroke in patients that recover from COVID-19: Comparisons to historical stroke prior to COVID-19 or stroke in patients with active COVID-19 infection
Source: PLoS One. 2022 Jun 24;17(6):e0270413. doi: 10.1371/journal.pone.0270413 (PMC9232148; doi:10.1371/journal.pone.0270413)
Supplement: Attachment — Submitted filename: Review Comments to the Author.docx [file pone.0270413.s001.docx]

Review Comments to the Author

Please use the space provided to explain your answers to the questions above. You may also include additional comments for the author, including concerns about dual publication, research ethics, or publication ethics. (Please upload your review as an attachment if it exceeds 20,000 characters)

Reviewer #1: The article is of scientific interest and in line with the purpose of the journal. The authors guidelines have been respected and the manuscript does not require an English language revision. There are some concerns that need to be addressed.

ABSTRACT
Purpose: Clearly specify the purpose of the study. "We compare characteristics" looks like you have compared and waited for something to come out.
Result: use abbreviations for groups.

**Response:** We have rewritten the abstract and taken into account the suggestions of the reviewer.

KEYWORDS
In order to increase the visibility of the article, do not use keywords already present in the title.

INTRODUCTION
"Introduction:" remove the ":".
The introduction is really too short both in terms of text and in terms of references (half a page is not acceptable also considering the importance of the journal).
You can take a cue and insert as a reference:
- Bai AV, Rabasco J, Ceccatelli V, Floridia S, Sbardella S, Petrignani C, Agostini F, Paoloni M. Suggestions for changes in professional procedures and adaptation to COVID-19: new models of care in the rehabilitation setting. Ann Ig. 2021 May-Jun; 33 (3): 299-304. doi: 10.7416 / ai.2021.2434.

- Agostini F, Mangone M, Ruiu P, Paolucci T, Santilli V, Bernetti A. Rehabilitation setting during and after Covid-19: An overview on recommendations. J Rehabil Med. 2021 Jan 5; 53 (1): jrm00141. doi: 10.2340 / 16501977-2776.

- Masiero S, Maccarone MC, Agostini F. Health resort medicine can be a suitable setting to recover disabilities in patients tested negative for COVID-19 discharged from hospital? A challenge for the future. Int J Biometeorol. 2020 Oct; 64 (10): 1807-1809. doi: 10.1007 / s00484-020-01947-4.

**Response:** We thank the reviewer for the valuable comment. We have rewritten the “Introduction” section and have considerably improved on the rationale for the value of the work we are presenting in the manuscript.

METHODS
The inclusion criteria and especially those of exclusion must be more specified.
Pay attention to the use of bold.
The character of the section titles (introduction ..) and subsections must be different.
"Analysis:" remove the ":".

**Response:**We are thankful to the reviewer. We have revised the “Methods” section and have added considerable details that are relevant to the “Results” presented in this manuscript

RESULTS
"Results:" remove the ":".
The results are written smoothly.

**Response:**We have provided additional information that we believe are of interest to the readership. We have added a figure that shows the outcomes in the three groups. This clearly shows the differences in outcome in patients with and without COVID-19 infection that suffer an acute stroke.

DISCUSSION
The discussion argues in an aderguta way for the results.

**Response:**The discussion section has been updated.

TABLES
Remove the color and adapt the font to the one required by the authors guidelines.
**Response:** Tables modified as suggested
FIGURE
The quality of the figure must be increased.
**Response:** We have added a new figure
REFERENCES
References need to be increased. Add the suggested references.

**Response:** Additional relevant references have been added

Reviewer #2: Dear Authors,
In this article you compared acute stroke in recovered, active COVID-19 and pre-pandemic patients in Qatar. It is an interesting article, but some issues have to been addressed. Moreover, Introduction and Results sections need a consistent improvement.

INTRODUCTION:
- It is insufficient. I suggest to the author to provide a background and summarize existing research with a deeper literature review. In detail, to introduce in general the stroke; to describe the alteration of cardiovascular system that could be linked with different stroke outcomes; and to introduce the importance of a global evaluation of the patient after COVID-19 infection, the disability related to long COVID-19, and the role of new technologies.
Accordingly, you also may cite:
- Vogrig A, Gigli GL, Bnà C, Morassi M. Stroke in patients with COVID-19: Clinical and neuroimaging characteristics. Neurosci Lett. 2021 Jan 19;743:135564. doi: 10.1016/j.neulet.2020.135564. Epub 2020 Dec 19. PMID: 33352277; PMCID: PMC7749733.
- Demeco et al. A. Rehabilitation of patients post-COVID-19 infection: a literature review. J Int Med Res. 2020 Aug;48(8):300060520948382. doi: 10.1177/0300060520948382. PMID: 32840156; PMCID: PMC7450453.
- Marotta et al. (2021), "Why is telerehabilitation necessary? A pre-post COVID-19 comparative study of ICF activity and participation", Journal of Enabling Technologies, Vol. 15 No. 2, pp. 117-121. https://doi.org/10.1108/JET-11-2020-0047
- “We have previously published on acute stroke in COVID-19 pandemic from Qatar.” It is not relevant for the reader.

**Response:** Please see comment to the first reviewer

METHODS
-“All acute stroke (AS) patients admitted to HGH between January-2019 to February-2020 were
evaluated for the study (PPS)”; “ 3111 patients admitted without COVID 19 in the 14 months prior to the pandemic (PPS)”. Did the patients result negative to COVID-19 test? I report that “The infection, caused by a coronavirus named SARS-CoV-2, was first recognised in Wuhan, China in December 2019” Dhillon P, Breuer M, Hirst N. COVID-19 breakthroughs: separating fact from fiction. FEBS J. 2020;287(17):3612-3632. doi:10.1111/febs.15442
- Did you make the power analysis?

**Response:** As per the suggestion, we have added considerable details.

RESULTS:
- “There was a significant decrease in stroke mimics in the CS cases as compared to PPS and RCS.” Please report p-value
- “and febrile on admission”; “ICU admission and intubated during admission”. Please check grammar

**Response:** We have made the appropriate corrections

TABLE
Table 1, please report all p-values.

**Response:** The ‘p-values are reported in the table

DISCUSSION.
- “Similar to numerous previous reports”. Please provide citations.
- “Cardioembolism was the final diagnosis in 14% of our patients with stroke following recovery from COVID-19 which is lower than the 25% seen in pre-COVID-19 cases and therefore likely did not contribute to the post-COVID-19 cases”. Is it significant? Please report p-value in results.
- “It is important to note that we noticed”. Please improve grammar.
- “This is similar to multiple observations from around the world.” Please report references.
- “A change over three months is brief and may not be sufficient to completely understand COVID-19-related changes.” What do you mean for change?

**Response:** We have made revisions to the “Discussion” sections
