# Supplementary material for: Ischemic stroke in patients that recover from COVID-19: Comparisons to historical stroke prior to COVID-19 or stroke in patients with active COVID-19 infection
Source: PLoS One. 2022 Jun 24;17(6):e0270413. doi: 10.1371/journal.pone.0270413 (PMC9232148; doi:10.1371/journal.pone.0270413)
Supplement: Attachment — Submitted filename: PLOS response to reviewers-March 02.docx [file pone.0270413.s002.docx]

Reviewer 1:
The manuscript must describe a technically sound piece of scientific research with data that supports the conclusions. The authors made all data underlying the findings in their manuscript fully available without restriction. However there are some issues about the language and grammar.

to authors: in the abstract the period of the study is between Jan 2020 and Feb 2021, while in the methods section in the manuscript you did not mention that only you mention that patients between January-2019 to February-2020 were evaluated for the study (PPS) and served as the reference comparator for the COVID-19 cases. # At the end of introduction (We also evaluated the characteristics of stroke in post-COVID-19 stroke to no history of COVID-19 infection) please consider revising language. # At results: male/female 2404 (73.7%)/4860 (26.3%)] please correct number. Why did not you exclude the stroke mimic patients from the clinical characteristics and risk factors studied? it would differ in the percentage of each studied parameter. # in discussion: (None of our patients had symptoms associated with long haulers and as best as best as we can determine) please correct. # (This is similar to our recent meta-analysis of multiple observations from around the world (ADD our recent meta-analysis) please put the reference. #(A change over three months is brief and may not be sufficient to completely understand COVID-19-related changes)this statement is not clear what do you mean by it?

**Response:** We thank the reviewer for the comments. We have corrected the time period and have removed the “stroke mimics” and done a complete re-analysis of the data for the revised manuscript. We have revised the abstract to reflect the changes. Additional changes in the “Introduction” and “Discussion” section will, we hope, improve the quality of the research.

Reviewer 2:
The timing of the manuscript is perfect given the ongoing pandemic. This study has provided new knowledge on stroke patients who have recovered from COVID-19. This is in addition to the already published data on acute stroke with acute Covid-19 infection.

However, there are a few minor comments

1) The authors should provide the population the HGH provides stroke services to since the hospital admits 95% of strokes in Qatar.

We have added information in the “Methods” section on the catchment area of HGH.

2) Statistics:
a) For consistency, p value should be added to “more frequently required intubation (CS:31.3%, PPS: 5.1% and RCS: 3.2%)” similar to the ICU admissions.

We apologize for this error. This was a typing error. We have added the p values to the above-mentioned variables.

b) P value for NIHSS, ICU admission and other three-group comparisons (p. 16-18 of PDF) – a single p value is reported, presumably reflecting the ANOVA; the post-hoc Bonferroni p values should also be reported, at least in supplementary

We thank the reviewer for the comment. The post-hoc Bonferroni analysis for the continuous variables are added to the results section

We apologize for this error. We have added the p values to the
3) The authors should remove or edit the comment “ ADD our recent meta-analysis” in the sentence “This is similar to our recent meta-analysis of multiple observations from around the world (ADD our recent meta-analysis) ( page 19 of PDF)

We apologize for the error. The correct manuscript/reference has been added

Reviewer 3:
The objective of this article was to compare characteristics and outcomes among acute stroke patients prior to the COVID-19 pandemic, stroke patients during active COVID-19 infection, and stroke patients after recovering from COVID-19. I appreciate the authors' revision of the manuscript. I believe this is an interesting article that would be of interest to the journals’ readers, but a number of improvements are still needed:

ABSTRACT
1. Some of the terminology should be more specific/clarified in the results section. For example: (1) What specific outcome do you mean by “poor stroke recovery”?, (2) “Large vessel disease and cardioembolic disease was significantly higher…”. Do you mean these were significantly more common?, (3) “There was a significant decline in stroke mimics…” Do you mean stroke mimics were less common, and if so, compared to which groups?
2. Methods: You mention that you included stroke cases between January 2020 and Feb 2021. However, in the full methods section, you stated that you included stroke patients admitted to HGH starting from January 2019. Please clarify

As noted in our response to reviewer 1, we have completely revised the abstract.

INTRODUCTION
1. “The number of COVID-19 worldwide…”. You should specify that this is the number of COVID-19 cases
2. While I appreciate the added detail in the introduction, make sure that everything is relevant to your study and it is clear why it is relevant for the reader. For example, what is the relevance of your prior publications that stroke occurs at a young age and acute stroke in COVID-19 pandemic from Qatar?
3. It doesn’t appear that the main objective was to evaluate the RISK of stroke (ie, the risk of having a stroke among all individuals in the source population who recovered from COVID-19- there is no quantification of this anywhere). Be careful with terminology.

We thank the reviewer for the comments. We have revised the “Introduction” section to make it more focused

METHODS
1. How did you define RCS patients? This should be clearly stated, because you need to define your main exposure group. Was it acute stroke within a specific number of days of recovering from COVID-19 infection? Would someone who had a stroke 1 year after recovering from COVID-19 be considered in the same group as individuals who had a stroke 1 month after recovering?
2. Statistical analysis: In your multivariate logistic regression model, did you also include a variable for the category of stroke (COVID-19 stroke, pre-COVID-19 stroke, recovered stroke)? Or was this model only run on patients with recovered stroke?

We thank the reviewer for the useful suggestion and we have made corrections accordingly

RESULTS
1. Under “demographic characteristics in the three groups”, the second and third sentences do not belong in the results section and should be moved to the discussion (interpretation of results should be limited to the discussion section only)
2. Same for the discussion about stroke mimics (“The details and possible reasons for the decrease in the percentage of stroke-mimics…”.. “Small vessel disease (SVD)…”)

We thank the reviewer for the comments and we have made appropriate changes

TABLE
1. Table 1- Should clarify that BMI is mean +/- standard deviation

We have changed it as suggested by the reviewer

Finally, please review the article thoroughly for typos.


[Note: HTML markup is below. Please do not edit.]

Reviewers' comments:

Reviewer's Responses to Questions

**Comments to the Author**

1. Is the manuscript technically sound, and do the data support the conclusions?

The manuscript must describe a technically sound piece of scientific research with data that supports the conclusions. Experiments must have been conducted rigorously, with appropriate controls, replication, and sample sizes. The conclusions must be drawn appropriately based on the data presented.

Reviewer #1: Yes

Reviewer #2: Yes

Reviewer #3: Yes

2. Has the statistical analysis been performed appropriately and rigorously?

Reviewer #1: N/A

Reviewer #2: Yes

Reviewer #3: Yes

3. Have the authors made all data underlying the findings in their manuscript fully available?

The [PLOS Data policy](http://www.plosone.org/static/policies.action#sharing) requires authors to make all data underlying the findings described in their manuscript fully available without restriction, with rare exception (please refer to the Data Availability Statement in the manuscript PDF file). The data should be provided as part of the manuscript or its supporting information, or deposited to a public repository. For example, in addition to summary statistics, the data points behind means, medians and variance measures should be available. If there are restrictions on publicly sharing data—e.g. participant privacy or use of data from a third party—those must be specified.

Reviewer #1: Yes

Reviewer #2: Yes

Reviewer #3: No

4. Is the manuscript presented in an intelligible fashion and written in standard English?

PLOS ONE does not copyedit accepted manuscripts, so the language in submitted articles must be clear, correct, and unambiguous. Any typographical or grammatical errors should be corrected at revision, so please note any specific errors here.

Reviewer #1: Yes

Reviewer #2: Yes

Reviewer #3: No

5. Review Comments to the Author

Please use the space provided to explain your answers to the questions above. You may also include additional comments for the author, including concerns about dual publication, research ethics, or publication ethics. (Please upload your review as an attachment if it exceeds 20,000 characters)
