# Supplementary material for: Ischemic stroke in patients that recover from COVID-19: Comparisons to historical stroke prior to COVID-19 or stroke in patients with active COVID-19 infection
Source: PLoS One. 2022 Jun 24;17(6):e0270413. doi: 10.1371/journal.pone.0270413 (PMC9232148; doi:10.1371/journal.pone.0270413)
Supplement: Attachment — Submitted filename: Response to the reviewers comments.docx [file pone.0270413.s003.docx]

Response to the reviewers comments:

the manuscript is technically sound and the data support the conclusions. However, the date of the study is not corrected in the abstract "We interrogated the Qatar stroke database for all stroke admissions between Jan 2020 and Feb 2021".

**Response: We thank the reviewer for our error. This has been corrected.**

in the methods section you stated that "The diagnosis of the stroke was within four weeks after full clinical and imaging from COVID-19 recovery" for patients with post- covid stroke while in the results section you stated that "The mean duration of time between recovery from COVID-19 infection and stroke was 126.9±75.9 days (median 124 days)".

**Response: We thank the reviewer and we have made the corrections in the method section.**

in discussion : "They were, however, more likely to be febrile on admission compared to pre-C" how can you explain fever in post -C stroke patients?

**Response: The reviewer raises a good point. We have discussed this with our Infectious Diseases colleagues and they can not offer any explanations for the higher rates of fiver in the post-COVID group. We have added a sentence in the ‘discussion’ section to clarify the issue.**

in discussion: " These patients had a rt-PCR confirmed diagnosis of COVID-17 infection" please correct.

**Response: We have made corrective edits**

"When comparing to the 1413 patients who had a stroke prior to the COVID-19 pandemic" please correct number of patients to 1324 for pre-covid stroke.

**Response: We thank the reviewer for the error and the correction has been made.**

in discussion: "It is also interesting that once the patients recovered, the stroke subtypes were very similar to what we had observed over the past 7 years (8)." what do you mean by stroke subtype? and did you compare with stroke patients in the previous 7 years or only previous 14 months as you mention in methods.

**Response: We have made appropriate editing in the ‘discussion’ section.**

at the end of discussion " Our data in 93 patients who had recovered from COVID-19 is reassuring in indicating no short-term effects of" please correct number of patients (43 not 93).

**Response: We have made the correction.**

**Reviewer # 3**

Thank you for addressing the reviewers' comments in your revised manuscript. I have no further substantial comments. However, please do a final review for typographical errors (there was a typo of "COVID-17" in the manuscript).

**We have made careful review of the manuscript. The error has been corrected.**
